# Identification of P2RY13 as an immune-related prognostic biomarker in lung adenocarcinoma: A public database-based retrospective study

**DOI:** 10.7717/peerj.11319

**Published:** 2021-05-05

**Authors:** Jiang Lin, Chunlei Wu, Dehua Ma, Quanteng Hu

**Affiliations:** Department of Thoracic Surgery, Taizhou Hospital of Zhejiang Province, Affiliated to Wenzhou Medical University, Taizhou, Zhejiang, China

**Keywords:** Lung adenocarcinoma, Tumor microenvironment, Immune, P2RY13

## Abstract

**Background:**

Lung adenocarcinoma (LUAD) is the leading histological subtype of non-small cell lung cancer (NSCLC).

**Methods:**

In the present study, the gene matrixes of LUAD were downloaded from The Cancer Genome Atlas to infer immune and stromal scores with the ‘Estimation of Stromal and Immune cells in Malignant Tumor tissues using Expression data’ (ESTIMATE) algorithm and identified immune-related differentially expressed genes (DEGs) between the high- and low-stromal/immune score groups. Next, all DEGs were subjected to univariate Cox regression and survival analyses to screen out prognostic biomarkers in the tumor microenvironment (TME), and were validated in the Gene Expression Omnibus database. Single-sample gene set enrichment analysis (ssGSEA) was performed to assess the level of tumor-infiltrating immune cells (TIICs) and immune functions, and GSEA was used to identified pathways altered by prognostic biomarkers.

**Results:**

Survival analysis showed that LUAD in the high-immune and stromal score group had a better clinical prognosis. A total of 303 immune-related DEGs were detected. Univariate Cox regression and survival analyses revealed that P2Y purinoceptor 13 (P2RY13) was a favorable factor for the prognosis of LUAD. ssGSEA and Spearman correlation analysis demonstrated that P2RY13 was highly correlated with various TIICs and immune functions. Several immune-associated pathways were enriched between the high- and low-expression P2RY13 groups.

**Conclusion:**

P2RY13 may be a potential prognostic indicator and is highly associated with the TME in LUAD. However, further experimental studies are required to validate the present findings.

## Introduction

Lung cancer is the most common malignancy globally. It was estimated that nearly 234,000 new cases would be diagnosed per year, and accounted for 13 and 14% of all new cancer cases in women and men, respectively (Barta, Powell & Wisnivesky, 2019; De Groot et al., 2018). Among them, ∼85% of patients were diagnosed with non-small cell lung cancer (NSCLC), which is mainly comprised of the adenocarcinoma histological type (65%) (Bray et al., 2018; Yu, Zhang & Zhang, 2020).

The immune system plays a vital role in the development and progression of malignant tumors (Chen et al., 2019b; Chen et al., 2019c; Kurbatov et al., 2020). Immunotherapy is a novel approved treatment for numerous tumors, which has revolutionized cancer treatment and has achieved satisfactory results. It acts through enhancing the function of the immune system to fight against tumors, and is highly associated with the tumor microenvironment (TME). The TME plays a vital role in the oncogenesis, progression and prognosis of cancer (Chen et al., 2019a). The TME is a complex system that consists of immune cells, stromal cells and extracellular matrix (Roma-Rodrigues et al., 2019). Of these, the immune and stromal cells are the most important components of the TME (Xiong et al., 2018). Stromal cells mainly consist of adipocytes, fibroblasts and mesenchymal stromal cells, while immune cells mainly comprise macrophages, natural killer cells and lymphocytes (Roma-Rodrigues et al., 2019). The immune cells in the TME could recognize malignant cells and eradicate cancer cells through immune surveillance (Corthay, 2014). However, in tumors, immune escape often occurs by avoiding recognition of tumor-associated antigens, which could facilitate the development, infiltration and metastasis of tumors (Lakshmi Narendra et al., 2013).

With the assistance of cytokines and chemokines, immune and stromal cells could regulate tumor behavior and influence the response of therapy. Numerous studies have shown that immune cells, stromal cells and immune-related biomarkers could be used as parameters for clinical decision, and assessment of therapeutic effect and prognosis. For example, Xu et al. (2020) reported that DEAH-box helicase 37, a biomarker highly associated with innate immune reactions and inflammation, impacted the prognosis of lung adenocarcinoma (LUAD) and the immune tolerance by activating the function of regulatory T cells and T cells. Yi et al. (2021) used 17 immune-related biomarkers to construct a prognostic signature for predicting the 3- and 5-year overall survival of patients with LUAD. The signature showed a good prediction performance. Additionally, the signature also could be used for the prediction and assessment of the efficacy of immunotherapy. Due to the aforementioned reasons, identifying prognostic biomarkers associated with TME immunity is important for the understanding and treatment of tumors.

Conventional detection technologies such as flow cytometry and immunohistochemistry are not capable of systematically obtaining consistent and accurate data of diverse immune and stromal cells simultaneously due to the restriction of the channel of markers (Zhou et al., 2019; Rohr-Udilova et al., 2018). Yoshihara et al., (2013) developed a novel tool called the ‘Estimation of Stromal and Immune cells in Malignant Tumor tissues using Expression data’ (ESTIMATE) algorithm for inferring the level of infiltrating stromal and immune cells through calculating the immune and stromal score. Several reports regarding glioma (Jia et al., 2018), colon cancer (Alonso et al., 2017), clear cell renal cell carcinoma (Chen et al., 2019b; Chen et al., 2019c) and breast cancer (Priedigkeit et al., 2017) have shown a good effectiveness of the ESTIMATE algorithm for calculating the immune and stromal score.

The present study applied the ESTIMATE algorithm to assess the gene expression profiles of LUAD obtained from The Cancer Genome Atlas (TCGA) (https://www.cancer.gov) to calculate immune and stromal scores. Next, differentially expressed genes (DEGs) between the high- and low-immune/stromal score groups were identified. The DEGs were then subjected to univariate Cox regression and survival analyses to screen prognostic immune-related biomarkers, which were validated in the Gene Expression Omnibus (GEO) dataset (https://www.ncbi.nlm.nih.gov).

## Material and Methods

### Data source and processing

The fragments per kilobase of transcript per million mapped reads level of gene-expression matrixes of patients with LUAD were obtained from TCGA. The raw data of the mRNA expression matrix of GSE68465 were collected from the GEO database and normalized with the ‘affy’ package in R 3.6.3 (https://www.r-project.org). The clinicopathological parameters of each patient were also downloaded. Cases lacking pathological diagnosis were excluded.

### ESTIMATE algorithm-derived immune and stromal scores

As described previously (Chen et al., 2019b; Chen et al., 2019c), the immune and stromal scores of each sample were calculated with the package ‘ESTIMATE’ in R. Next, the scores of patients in TCGA dataset were evaluated with the package ‘survminer’ to infer the optimal cut-off value, which divided patients into high- or low-immune/stromal score groups. Kaplan–Meier plot and log-rank test were performed to construct a survival curve to illustrate the association of immune/stromal scores and overall survival of patients with LUAD. Patients in the GEO dataset were classified into high- or low-immune/stromal score groups according to the optimal cut-off value, and survival analysis was performed.

### Expression analysis of DEGs

Using | log fold-change (FC) | >1.2 and false discovery rate (FDR) <0.05 as the criteria, the Bioconductor package ‘edgeR’ was used to determine DEGs between high- and low-immune/stromal score groups in TCGA dataset. The overlapping DEGs were utilized for further analysis.

### Functional enrichment analysis and protein-protein interaction (PPI) network construction

All the overlapping DEGs were used for Kyoto Encyclopedia of Genes and Genomes (KEGG) and Gene Ontology (GO) analyses. FDR < 0.05 was set as the threshold. In addition, a PPI network of all the overlapping DEGs was obtained from Search Tool for the Retrieval of Interacting Genes/Proteins (https://string-db.org) with a confidence >0.9 as the threshold, and was reconstructed with Cytoscape version 3.6 (https://cytoscape.org).

### Identification of prognostic immune-related biomarkers

All the overlapping DEGs in TCGA dataset were subjected to univariate Cox regression analysis to identify prognosis-related genes. Survival analysis was applied to compare the survival difference between high- and low-expression of DEGs. Similarly, univariate Cox regression and survival analyses of DEGs were also performed in the GEO dataset to validate the result in TCGA dataset. Genes meeting the criteria (univariate Cox regression analysis, *P* < 0.01; survival analysis, *P* < 0.05) in both datasets were selected as prognostic biomarkers for further research.

Group comparisons of the expression levels of biomarkers among different clinical characteristics were performed with Student’s *t*-test. Univariate and multivariate Cox regression models were conducted to identify whether the expression of biomarkers was an independent prognostic factor for LUAD.

### Single-sample gene set enrichment analysis (ssGSEA)

ssGSEA was performed to assess the level of tumor-infiltrating immune cells (TIICs) and immune functions with the BiocManager package ‘Gene Set Variation Analysis’. The method for ssGSEA was based on a rank value of each gene, which defined a score representing the degree of absolute enrichment of a particular gene set in each sample. In total, 28 specific gene sets (15 immune cell gene sets and 13 immune function gene sets) were acquired from other studies (Charoentong et al., 2017; Shi et al., 2020; Zuo et al., 2020). The association of biomarkers with TIICs and immune functions were investigated with Spearman correlation analysis.

### Association of biomarkers with immunomodulators and patients’ response to immunotherapy

In the present study, several key immunomodulators [cytotoxic T-lymphocyte-associated protein 4 (CTLA-4), intercellular adhesion molecule 1, inducible T-cell costimulatory, interferon- *γ*, lymphocyte activating gene 3, T cell immunoreceptor with Ig and ITIM domains, natural killer gene 2A, programmed cell death protein 1 (PD-1), programmed death-ligand 1 (PD-L1), T-cell immunoglobulin and mucin-domain containing-3 and V-domain Ig suppressor of T cell activation] were quantified. Student’s *t*-test and Spearman correlation analysis were performed to determine the association of immunomodulators and immune/stromal scores as well as prognostic biomarkers.

The Cancer Immunome Atlas (https://tcia.at/) is a public database, which analyzes next-generation sequencing data to present immune landscapes and anti-genomes of 20 solid tumors, and calculates the immunophenoscore (IPS) (Charoentong et al., 2017). The IPS value is ranked from 0 to 10, and is positively correlated with tumor immunogenicity. Furthermore, the IPS could reflect the response to immune checkpoint inhibitors treatment. The present study analyzed two types of IPS values (IPS: PD-1/PD-L1/PD-L2 blocker and IPS: CTLA-4 blocker) to investigate the different responses to anti-PD-1/PD-L1 and anti-CTLA-4 treatment between patients with low- and high-stromal score/immune score/P2Y purinoceptor 13 (P2RY13) level with Wilcoxon signed-rank test.

### GSEA

To determine the potential signaling pathways altered by prognostic biomarkers, GSEA was performed with FDR<0.05 as the threshold.

## Results

### Patient cohorts

**Figure 1:**
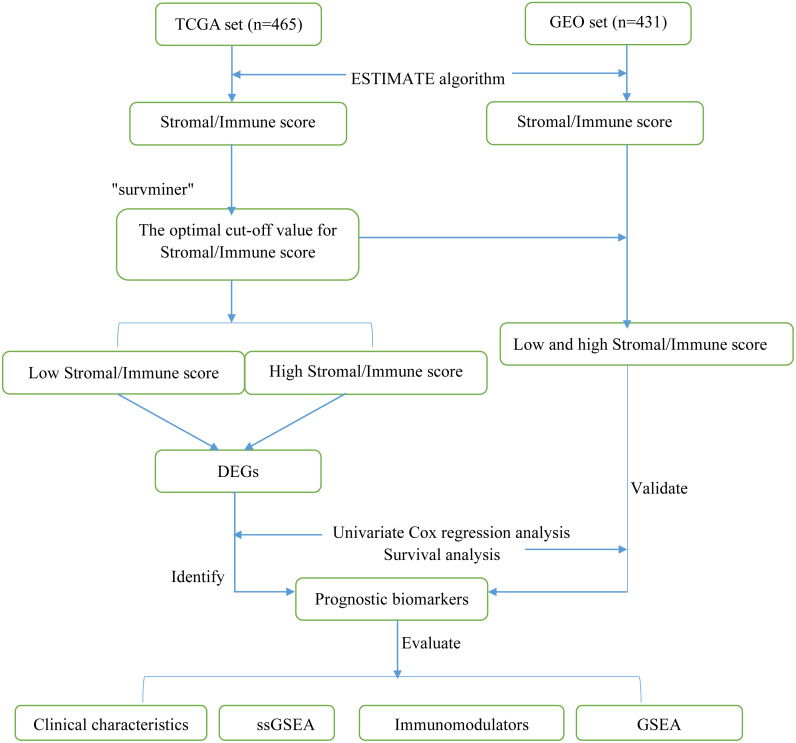
Flow diagram of the present study. TCGA, The Cancer Genome Atlas; GEO, Gene Expression Omnibus; DEGs, differentially expressed genes; ssGSEA, single-sample gene set enrichment analysis.

A total of 896 LUAD samples (465 samples in TCGA dataset and 431 samples in the GEO dataset) were identified. The detailed demographic and baseline characteristics of these 896 patients with LUAD are presented in Table 1. The flow diagram of the present study is shown in Fig. 1.

**Table 1:** The baseline characteristics of lung adenocarcinoma patients in this study.

Parameter	TCGA set	GEO set
Gender		
Female	254(54.62%)	216(50.12%)
Male	211(45.38%)	215(49.88%)
Age		
<65	232(49.89%)	226(52.44%)
≥65	233(50.11%)	205(47.56%)
TNM stage		
I	261(56.12%)	270(62.65%)
II	106(22.80%)	100(23.20%)
III	74(5.92%)	61(14.15%)
IV	84(18.06%)	0
Tumor size		
T1	159(34.19%)	145(33.64%)
T2	248(53.33%)	244(56.61%)
T3	40(8.60%%)	27(6.73%)
T4	18(3.87%)	11(3.02%)
Lymph node		
N0	309(66.45%)	292(67.75%)
N1-3	156(33.55%)	139(32.25%)
Metastasis		
M0	441(94.84%)	431(100%)
M1	24(5.16%)	0
EGFR mutation		
No	174(37.42%)	0
Yes	69(15.05%)	0
NA	221(47.53%)	431(100%)
KRAS mutation		
No	34(7.32%)	0
Yes	17(3.66%)	0
NA	414(89.02%)	431(100%)
Stromal score		
Low	178(38.28%)	95(22.04%)
High	287(61.72%)	336(77.96%)
Immune score		
Low	294(63.23%)	323(74.94%)
High	171(36.77%)	108(25.06%)
Survival status		
Alive	310(66.67%)	202(46.87%)
Dead	155(33.33%)	229(53.13%%)
Total	465(100%)	431(100%)

**Notes.**

Abbreviations: TCGA The Cancer Genome Altas GEO Gene Expression Omnibus NA represents information not available

### Evaluation of immune and stromal scores

**Figure 2:**
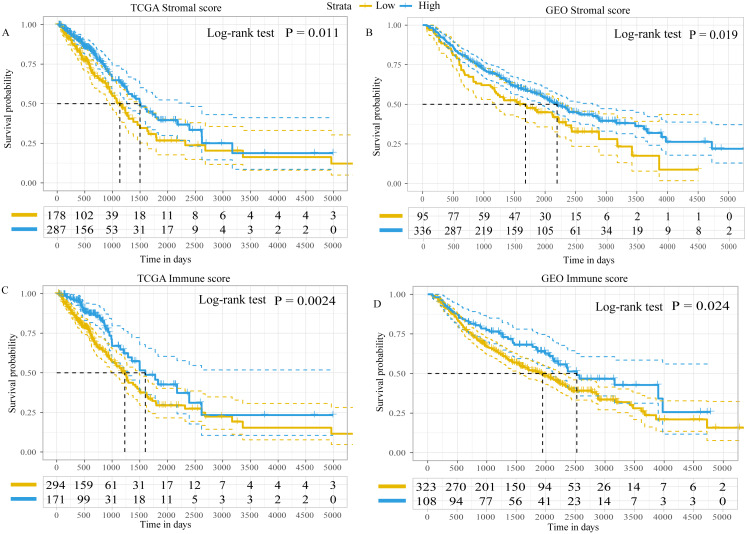
Kaplan–Meier plot revealed the prognostic value of Stromal scores and Immune score. Kaplan–Meier plot revealed the prognostic value for overall survival of (A) stromal scores in TCGA dataset, (B) stromal scores in the GEO dataset, (C) immune scores in TCGA dataset and (D) immune scores in the GEO dataset. The difference in survival was compared with the log-rank test. *P* < 0.05 was considered to indicate a statistically significant difference. TCGA, The Cancer Genome Atlas; GEO, Gene Expression Omnibus.

Using the ESTIMATE algorithm, the present study calculated the immune and stromal scores of patients in both the GEO and TCGA datasets. The optimal cut-off value for immune score and stromal score was 1,795.5 and 13.5, respectively, which divided patients into high- and low-immune/stromal score groups. Survival analysis in TCGA dataset demonstrated that the prognosis of patients with LUAD with high-stromal/immune scores was better than that of patients with low-stromal/immune scores (Figs. 2A and 2C), which was similar to the results obtained in the GEO dataset (Figs. 2B and 2D).

In addition, the immune scores were significantly different among different tumor-node-metastasis (TNM) stages and tumor sizes in both TCGA dataset (TNM stage, *P* = 0.039; tumor sizes, *P* < 0.001; Fig. S1) and the GEO dataset (TNM stage, *P* = 0.046; tumor sizes, *P* = 0.045; Fig. S2). The stromal scores were significantly different among tumor sizes in the GEO dataset (*P* = 0.014; Fig. S2), whereas in TCGA dataset there were no differences (*P* = 0.210; Fig. S1). There was no statistically significant difference in immune or stromal scores between patients who were < 65 and ≥65 years of age; between female and male patients; between patients with and without lymph node metastasis; between patients with and without distant metastasis; or between patients with and without epidermal growth factor receptor (EGFR)/KRAS proto-oncogene, GTPase (KRAS) mutation (data not shown). Furthermore, no difference in stromal scores was observed among different TNM stages.

### Functional enrichment analysis and PPI network

**Figure 3:**
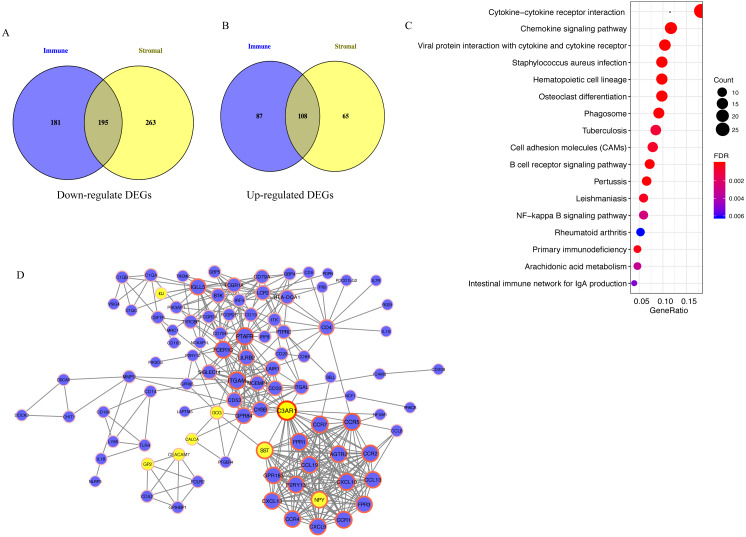
Analysis of 303 overlapping DEGs. Analysis of 303 overlapping DEGs, including (A) 195 downregulated and (B) 108 upregulated overlapping DEGs. (C) Kyoto Encyclopedia of Genes and Genomes analysis of DEGs. (D) Protein-protein interaction network with confidence >0.9. Blue and yellow nodes represent upregulated and downregulated genes, respectively. DEGs, differentially expressed genes.

A total of 195 downregulated and 108 upregulated overlapping DEGs were identified between the high- and low-immune/stromal score groups in TCGA dataset (Fig. 3A). Next, KEGG and GO analyses were performed to illustrate the role of 303 overlapping DEGs. KEGG analysis demonstrated that 17 pathways were enriched in these DEGs, including ‘Cytokine-cytokine receptor interaction’, ‘Chemokine signaling pathway’ and ‘B cell receptor signaling pathway’, which were highly associated with the immune system (Fig. 3B). In GO analysis, 34 terms (four terms of molecular function, five terms of cellular component and 25 terms of biological processes) were identified (Table S1).

For exploring the interactions among 303 overlapping DEGs, a PPI network was constructed, which consisted of 98 nodes (90 upregulated and 8 downregulated DEGs) and 395 edges (Fig. 3C).

### P2RY13 is an immune-related prognostic biomarker

**Figure 4:**
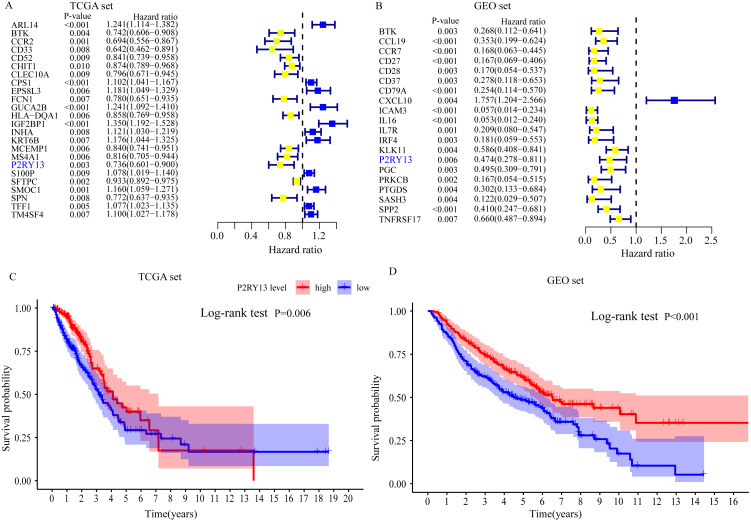
Identification of a prognostic biomarker. (A and B) Genes with *P* < 0.01 in univariate Cox regression analysis in (A) TCGA and (B) GEO datasets. (C and D) Survival analysis of P2Y purinoceptor 13 in (A) TCGA and (D) GEO datasets. The difference in survival was compared with the log-rank test. *P* < 0.05 was considered to indicate a statistically significant difference. TCGA, The Cancer Genome Atlas; GEO, Gene Expression Omnibus.

Univariate Cox regression and survival analyses were applied to determine the best prognosis-related genes. The results of the univariate Cox regression analysis showed that, among 303 DEGs, 24 genes in TCGA dataset and 20 genes in the GEO dataset exhibited *P* < 0.001 (Fig. 4A and 4B). In addition, survival analysis demonstrated that 30 genes in TCGA dataset and 21 genes in the GEO dataset had an important effect on the prognosis of LUAD (data not shown). Subsequently, immune-related prognostic biomarkers were identified with the criteria univariate Cox regression analysis, *P* < 0.01 and survival analysis, *P* < 0.05 in both sets, and it was found that only one gene (P2RY13) met the aforementioned criteria: Univariate Cox regression analysis: TCGA dataset, hazard ratio (HR) = 0.736, 95% confidence interval (CI) = 0.601–0.900, *P* = 0.003 (Fig. 4A) and GEO dataset, HR = 0.474, 95% CI [0.278–0.811], *P* = 0.006 (Fig. 4B); and survival analysis: TCGA dataset, *P* = 0.006 (Fig. 4C) and GEO dataset, *P* < 0.001 (Fig. 4D). Therefore, P2RY13 was selected as a prognostic biomarker for further investigation.

### Correlation between P2RY13 and clinical characteristics

**Figure 5:**
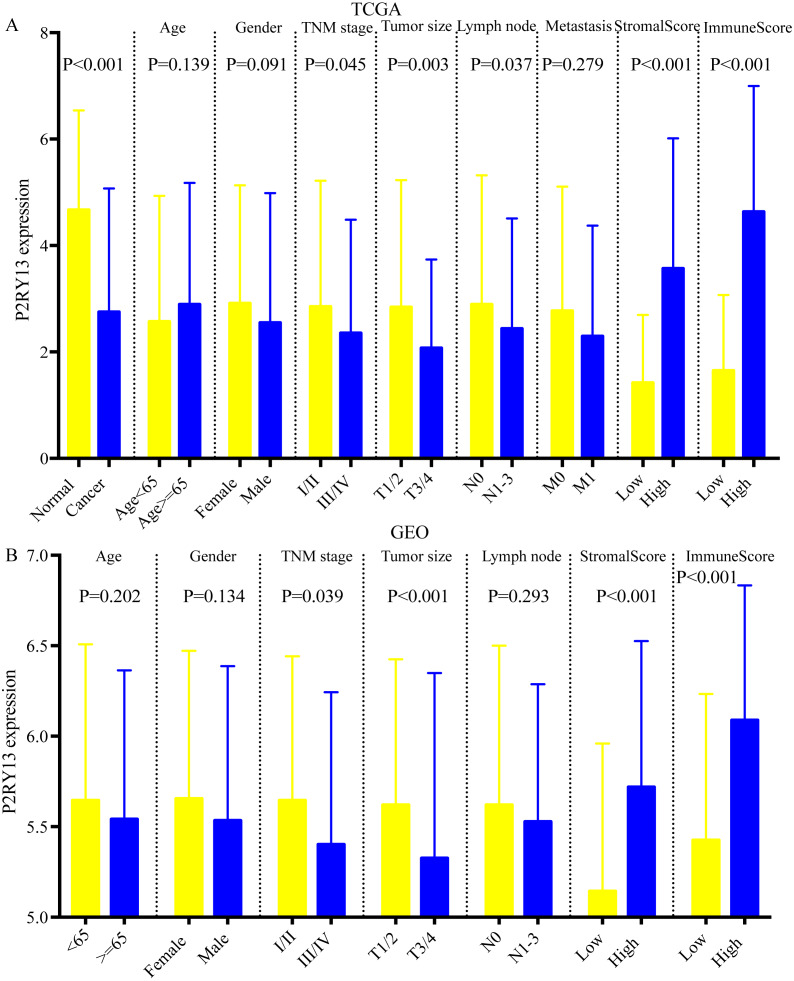
Correlation between P2RY13 and clinical characteristics. Correlation between P2Y purinoceptor 13 and clinical characteristics in (A) The Cancer Genome Atlas and (B) Gene Expression Omnibus datasets. The data are presented as the mean ± standard deviation, and were compared with Student’s *t*-test. *P* < 0.05 was considered to indicate a statistically significant difference.

As shown in Figs. 5A and 5B, the expression of P2RY13 in patients with high stromal/immune score was significantly upregulated in TCGA dataset (stromal score, *P* < 0.001; immune score, *P* < 0.001), which was in agreement with the results obtained in the GEO dataset (stromal score, *P* < 0.001; immune score, *P* < 0.001). In both datasets, patients with stage I/II had higher P2RY13 expression level (TCGA dataset, *P* = 0.045; GEO dataset, *P* = 0.039). Similar results were observed in patients with T1/T2. In addition, the P2RY13 expression level of patients with lymph node metastasis in TCGA dataset was significantly decreased (*P* = 0.037). However, in the GEO dataset, there was no difference between patients with or without lymph node metastasis (*P* = 0.293). In TCGA dataset, no difference was observed between patients with or without EGFR mutation (Fig. S3A). A similar phenomenon was found in patients with or without KRAS mutation (Fig. S3A).

Univariable Cox regression analysis in two datasets revealed that the expression level of P2RY13 was a meaningful factor influencing the prognosis of patients with LUAD (TCGA dataset, HR = 0.615, 95% CI [0.433–0.873], *P* = 0.006; GEO dataset, HR=0.631, 95% CI=0.487–0.819, *P* < 0.001) (Table 2). In addition, multivariable Cox regression analysis indicated that P2RY13 was an independent prognosis-related factor (TCGA dataset, HR = 0.601, 95% CI [0.423–0.856], *P* = 0.005; GEO dataset, HR = 0.760, 95% CI [0.582–0.994], *P* = 0.045) (Table 2).

**Table 2:** Univariate and multivariate Cox regression analysis in TCGA and GEO set.

		univariate Cox regression		multivariate Cox regression	
Covariate	No,	HR(CI 95%)	*P*	HR(CI 95%)	*P*
**TCGA set**					
Age			0.236		0.059
<65	232	reference		reference	
≥65	233	1.214(0.881–1.674)		1.375(0.989–1.913)	
Gender			0.561		0.741
Female	254	reference		reference	
Male	211	1.098(0.801–1.506)		0.947(0.687–1.306)	
TNM stage			<0.001		0.500
I/II	367	reference		reference	
III/IV	158	2.617(1.881–3.643)		1.177(0.733–1.888)	
Tumor size			<0.001		0.037
T1/T2	407	reference		reference	
T3/T4	58	2.424(1.608–3.654)		1.637(1.030–2.601)	
Lymph node			<0.001		<0.001
N0	309	reference		reference	
N1-3	156	2.872(2.090–3.946)		2.533(1.717–3.737)	
Metastasis			0.007		0.197
M0	441	reference		reference	
M1	24	2.086(1.222–3.560)		1.498(0.811–2.766)	
P2RY13			0.006		0.005
Low	232	reference		reference	
High	233	0.615(0.433–0.873)		0.601(0.423–0.856)	
**GEO set**					
Age			0.032		0.053
<65	226	reference		reference	
≥65	205	1.331(1.024–1.729)		1.300(0.996–1.697)	
Gender			0.010		0.164
Female	216	reference		reference	
Male	215	1.410(1.084–1.833)		1.215(0.924–1.598)	
TNM stage			<0.001		0.022
I/II	370	reference		reference	
III/IV	61	3.547(2.595–4.848)		1.628(1.072–2.474)	
Tumor size			<0.001		<0.001
T1/T2	389	reference		reference	
T3/T4	38	3.293(2.264–4.787)		2.299(1.520–3.478)	
Lymph node			<0.001		<0.001
N0	292	reference		reference	
N1-3	139	2.747(2.107–3.568)		2.099(1.501–2.935)	
P2RY13			<0.001		0.045
Low	215	reference		reference	
High	216	0.631(0.487–0.819)		0.760(0.582–0.994)	

**Notes.**

Abbreviations: TCGA The Cancer Genome Altas GEO Gene Expression Omnibus HR hazard ratios CI confidence interval

Next, the expression of P2RY13 at the protein level was investigated with the online database The Human Protein Atlas (https://www.proteinatlas.org). The results of immunohistochemistry showed that the representative protein expression of P2RY13 in LUAD tissues was downregulated (Figs. S3C and S3D). The prognostic value of P2RY13 at the protein level was explored with Clinical Proteomic Tumor Analysis Consortium (National Cancer Institute; https://proteomics.cancer.gov/programs/cptac), and the results revealed that a high P2RY13 protein level predicted improved prognosis (*P* = 0.021; Fig. S3B).

### P2RY13 is associated with TIICs and immune functions

**Figure 6:**
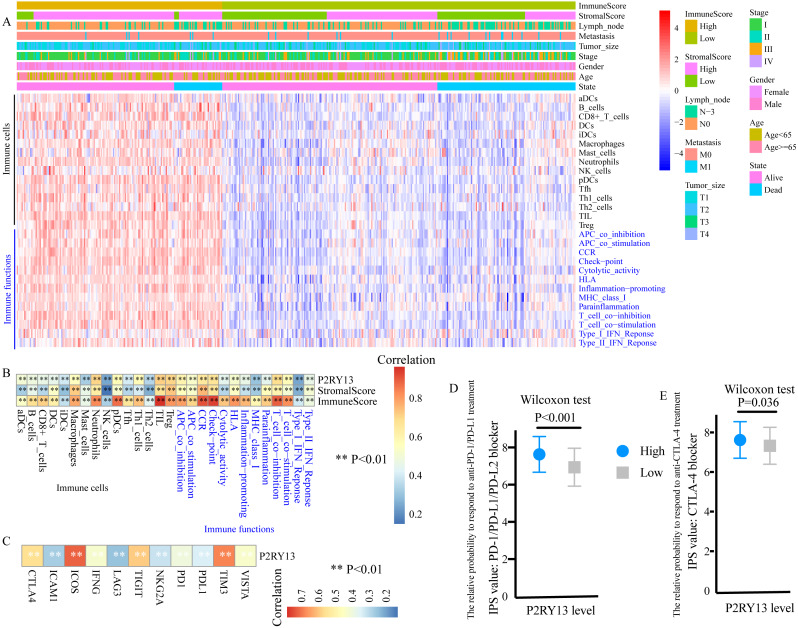
P2RY13 is associated with tumor-infiltrating immune cells, immune functions and immunomodulators. (A) Distribution of 28 gene sets, including 15 immune cell gene sets and 13 immune function gene sets. (B) Spearman correlation analysis revealed the correlation of P2RY13 as well as immune and stromal scores with 28 gene sets. (C) Spearman correlation analysis revealed the correlation of P2RY13 with 11 immunomodulators. (D and E) Relative probabilities to respond to anti-programmed cell death protein 1/programmed death-ligand 1 and anti-cytotoxic T-lymphocyte-associated protein four treatment in patients with lung adenocarcinoma with high and low P2RY13 expression. The data are presented as the mean standard deviation, and were compared with Wilcoxon signed-rank test. aDCs, activated dendritic cells; iDCs, immature DCs; pDCs, plasmacytoid DCs; Tfh, T follicular helper cells; Th1, type 1 helper; Th2, type 2 helper; TILs, tumor-infiltrating lymphocytes; Tregs, regulatory T cells; HLA, human leukocyte antigen; CCR, C-C chemokine receptor; APCs, antigen presenting cells; MHC, major histocompatibility complex; IPS, immunophenoscore; P2RY13, P2Y purinoceptor 13.

ssGSEA was performed to evaluate the level of TIICs and immune functions. The distribution of 28 gene sets, including 15 immune cell gene sets and 13 immune function gene sets, is presented in Fig. 6A. Spearman correlation analysis revealed a strong positive correlation between the expression level of P2RY13 and 28 gene sets (Fig. 6B). In addition, the association between the immune and stromal scores and these 28 gene sets was statistically significant (Fig. 6B).

### Association of P2RY13 with immunomodulators and patients’ response to immunotherapy

The present study quantified 11 immunomodulators, all of which were significantly upregulated in the high-stromal/immune score group (Fig. S4A and S4B). Furthermore, P2RY13 was positively correlated with all the 11 immunomodulators (Fig. 6C).

Next, the responses to anti-PD-1/PD-L1 and anti-CTLA-4 treatment among different groups were explored. Both the IPS: PD-1/PD-L1/ PD-L2 blocker and the IPS: CTLA-4 blocker were higher in patients with LUAD with high P2RY13 expression level (Figs. 6D and 6E), indicating that the relative probabilities to respond to anti-PD-1/PD-L1 and anti-CTLA-4 treatment were higher in patients with high P2RY13 expression level. Similar results were observed in patients with high-stromal/immune score (Figs. S4C and S4D).

### GSEA

**Figure 7:**
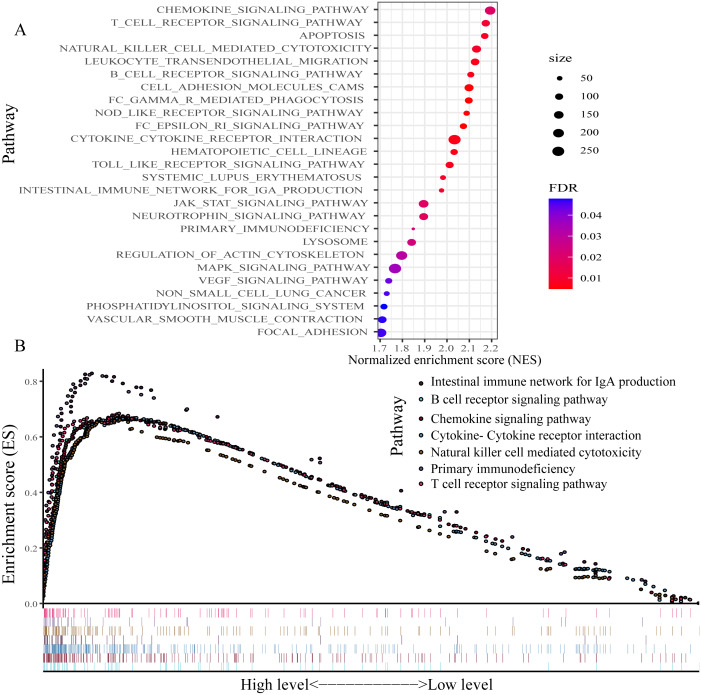
Gene set enrichment analysis between patients with high and low P2RY13 expression. (A) A total of 26 Kyoto Encyclopedia of Genes and Genomes pathways were enriched. (B) In total, seven immune-related pathways were enriched in patients with high P2RY13 expression level. P2RY13, P2Y purinoceptor 13.

For exploring the changes in KEGG pathways between the high- and low-expression level of P2RY13 groups, GSEA analysis was performed. The result indicated that 26 pathways were enriched (Fig. 7A). Of note, in patients with high P2RY13 expression, a few immune-related pathways were identified, including ‘B cell receptor signaling pathway’, ‘T cell receptor signaling pathway’, ‘Intestinal immune network for IgA production’, ‘Natural killer cell-mediated cytotoxicity’, ‘Primary immunodeficiency’, ‘Chemokine signaling pathway’ and ‘Cytokine-Cytokine receptor interaction’ (Fig. 7B).

## Discussion

The TME is a regulatory factor in the tumorigenesis, progression and prognosis of cancer, which consists of various types of cells and cellular components (Roma-Rodrigues et al., 2019). Previous studies have demonstrated that immune and stromal cells can markedly affect tumor progression and response to treatment, and are able to predict prognosis (Chen et al., 2019b; Chen et al., 2019c; Maekawa et al., 2008). Understanding the changes in the TME, and the identification of biomarkers in the TME may contribute to the development of novel strategies for diagnosis, therapy and prognosis assessment.

In the current study gene matrixes of LUAD were downloaded from the GEO and TCGA databases, and the ESTIMATE algorithm was applied to infer the infiltrating immune and stromal cells in the TME by calculating immune and stromal scores. The results indicated that patients with LUAD with high immune/stromal scores had improved clinical outcomes than those with low immune/stromal scores.

Next, TME-related DEGs were identified between the high- and low-stromal/immune score groups, which may contribute to the changes in the TME. A total of 303 overlapping DEGs were identified. Functional enrichment analysis revealed that 17 pathways were enriched in those 303 DEGs, including ‘Cytokine-cytokine receptor interaction’, ‘Chemokine signaling pathway’, ‘B cell receptor signaling pathway’, ‘Primary immunodeficiency’ and other immune-related pathways, indicating that those 303 DEGs were highly associated with immunity and immune function. Next, the 303 overlapping DEGs were subjected to univariate Cox regression and survival analyses to determine potential prognostic biomarkers in the TME. The results demonstrated that patients with LUAD with low P2RY13 expression level exhibited worse clinical outcomes than those of patients with high P2RY13 expression level, indicating that P2RY13 may be a potential TME-related biomarker for estimating the clinical outcome of LUAD. Furthermore, it was found that, in both datasets, the expression level of P2RY13 in patients with at III/IV stage and T3/4 was significantly downregulated. In addition, the expression level of P2RY13 in patients with lymph node metastasis in TCGA dataset was significantly decreased. However, there was no difference in P2RY13 expression between patients with and without lymph node metastasis in the GEO dataset. Univariate and multivariate Cox regression analyses demonstrated that P2RY13 was an independent prognosis-related factor for LUAD. The aforementioned results indicated that P2RY13 was a key factor influencing the development and prognosis of LUAD. It was also identified that P2RY13 was upregulated in the high-stromal/immune score group, and it was highly associated with the infiltration of various immune cells in the TME and the expression of several immunomodulators. In addition, numerous immune-associated pathways were enriched in patients with LUAD with high P2RY13 level, indicating that P2RY13 may produce a marked effect on LUAD through influencing the TME and tumor immunization.

P2RY13 is a G protein-coupled receptor that responds to extracellular purine and pyrimidine nucleotides, and is involved in the negative regulation of adenylate cyclase activity (Pérez-Sen et al., 2017). Purinergic receptors (PRs) consist of P1Rs (A1, A2A, A2B and A3) and P2Rs (P2RY1, 2, 4, 6 and 11-14, and P2RX1-7) (Graner, 2018). P2Rs participate in the metabolism of extracellular ATP, which is a damage-associated molecular pattern and the main source of adenosine in the TME (Graner, 2018; Jacob et al., 2013). High levels of extracellular ATP generate an inflammatory environment in the tumor, which enhances tumor progression and inhibits immune cells, and ultimately represses tumor antigen presentation, influences the populations and functions of immune cells, and inhibits antitumor immunity (Burnstock, 2017; Dwyer, Kishore & Robson, 2020). In addition, previous studies suggested that purinergic signaling could modulate energy metabolism and several intracellular trophic pathways to regulate tumor growth, invasion and metastasis (Burnstock, 2017; Dwyer, Kishore & Robson, 2020).

Several studies have reported that P2RY13 was a key regulator of cholesterol transport and hepatic high-density lipoprotein endocytosis (Jacquet et al., 2005; Fabre et al. 2010), and was involved in bone formation and remodeling (Pérez-Sen et al., 2017), as well as in cell survival and neuroprotection (Pérez-Sen et al., 2015). Animal experiments showed that P2RY13 could protect hosts from viral infections, indicating that P2RY13 may be associated with inflammatory and immune reactions (Zhang et al., 2019; Shen et al., 2020). Additionally, P2RY13 was shown to bind to Ca^2+^ to mediate the release of several pro-inflammatory cytokines in the microglia and astrocytes, which are the main immune cells in the central nervous system, thus preventing the proliferation of astroglia (Quintas et al., 2018). The present study found that the expression level of P2RY13 in LUAD tissues was downregulated. However, previous studies reported that P2RY13 expression in acute myeloid leukemia was increased, and regulated cyclic adenosine monophosphate-mediated cytarabine resistance (Aroua et al., 2020; Maiga et al., 2016). To date, there is no basic research on the role of P2RY13 in LUAD, nor studies on the role of P2RY13 on the TME or immune reactions in LUAD. Therefore, additional in vitro and in vivo experiments are required.

The present study demonstrated that P2RY13 was a favorable factor for the prognosis of patients with LUAD, which is in line with previous findings (Li et al., 2018; Fan et al., 2020). Li et al. (2018) investigated the prognostic value of several pyrimidine metabolic rate-limiting enzymes, and found that P2RX1, P2RX7, P2RY12, P2RY13 and P2RY14 were highly associated with the overall survival of patients with LUAD. In the present study, in addition to finding the prognostic value of P2RY13, its association with immunological functioning was also explored, and it was found that P2RY13 was a prognostic factor for LUAD and it was highly associated with the immune system in LUAD. TCGA database was used in a previous study to identify 374 DEGs between high- and low-immune/stromal score groups with FDR<0.05 as the criterium, and 4 prognostic DEGs [C-C motif chemokine receptor (CCR)2, CCR4, P2RY12 and P2RY13]. The intersection of the top 30 genes in the PPI network and genes with *P* < 0.05 in univariate Cox regression analysis were selected. By contrast, the present study used —log FC—>1.2 and FDR<0.05 as the criteria to determine 303 DEGs between high- and low-immune/stromal score groups, and *P* < 0.01 in univariate Cox regression analysis as well as *P* < 0.05 in survival analysis were the criteria set to screen immune-related prognostic biomarkers. In addition, the results were validated in the GEO dataset, and it was found that only P2RY13 met the aforementioned criteria in both datasets.

The present study has certain limitations. First, the present study is a retrospective study, and all the cases were retrospective samples. Thus, validation in prospective samples is required. Second, all the samples were collected from a public database. In addition, in the GEO dataset, no stage-IV patients were enrolled, which may lead to potential selection bias. Therefore, additional LUAD cases, particularly stage-IV patients, are needed. Third, although the relative probabilities to respond to anti-PD-1/PD-L1 and anti-CTLA-4 treatment were predicted to be higher in patients with high P2RY13 expression levels, there was no treatment-related data presented. Therefore, data on treatment is necessary. Finally, the association of P2RY13 with immune cells and immune function was investigated by bioinformatics, which revealed that P2RY13 was highly associated with several immune cells and immune functions. However, certain immune cells and immune functions were pro-tumor, and various were antitumor. Thus, additional basic and clinical studies are required to explore and validate the role of P2RY13 in immune cells and the immune system.

In summary, the ESTIMATE algorithm was applied in the present study to infer the immune and stromal scores of patients with LUAD, which were highly associated with the prognosis of LUAD. In addition, P2RY13 was identified as a potential prognostic indicator, which was highly associated with the TME in LUAD. However, additional in vitro and in vivo experiments are required to validate the present findings.

##  Supplemental Information

10.7717/peerj.11319/supp-1 Table S1 Go analysis of 303 overlapping DEGs

10.7717/peerj.11319/supp-2 Figure S1 Distribution of immune and stromal score in TCGA set The data were compared with *T*-test. *P* < 0.05 was set as the threshold.

10.7717/peerj.11319/supp-3 Figure S2 Distribution of immune and stromal score in GEO set The data were compared with *T*-test. *P* < 0.05 was set as the threshold.

10.7717/peerj.11319/supp-4 Figure S3 The analysis of P2RY13 at protein level (A) Comparison of P2RY13 expression between patients with and without EGFR/KRAS mutation. The data were presented as mean ±  SD (standard deviation), and compared with *T*-test. (B) The prognostic value of P2RY13 at protein level. The survival difference was compared with log-rank test. *P* < 0.05 was set as the threshold. (C–D) The results of Immunohistochemistry showed the representative protein expression of P2RY13 in LUAD tissues was down-regulated.

10.7717/peerj.11319/supp-5 Figure S4 The expression of immunomodulators The expression of immunomodulators (A) between high- and low- stromal score group, (B) between high- and low- immune score group. The data were presented as mean±  SD (standard deviation), and compared with *T*-test. (C–D) The relative probabilities to respond to anti-PD-1/PD-L1 and anti-CTLA-4 treatment in LUAD patients with high and low stromal/immune expression. The data were presented as mean ± SD (standard deviation), and compared with Wilcoxon test. *P* < 0.05 was set as the threshold.

## Abbreviations

LC: Lung cancer
NSCLC: Non-small cell lung cancer
LUAD: lung adenocarcinoma
TCGA: The Cancer Genome Altas
GEO: Gene Expression Omnibus
TME: The tumor microenvironment
TIICs: tumor-infiltrating immune cells
GSEA: Gene set enrichment analysis
ESTIMATE: Estimation of Stromal and Immune cells in Malignant Tumour tissues using Expression data
FDR: False discovery rate
PPI: protein-protein interaction
KEGG: Kyoto Encyclopedia of Genes and Genomes
GO: Gene Ontology
ssGSEA: Single-sample gene set enrichment analysis
HR: hazard ratios
CI: confidence interval
DEGs: differentially expressed genes
IPS: immunophenoscore

## Additional Information and Declarations

### Data Availability

The following information was supplied regarding data availability: All data were gathered from the TCGA (The Cancer Genome Altas, TCGA-LUAD, https://cancergenome.nih.gov) and GEO (Gene Expression Omnibus, https://www.ncbi.nlm.nih.gov/geo/): GSE68465.
