# Supplementary material for: Identification of P2RY13 as an immune-related prognostic biomarker in lung adenocarcinoma: A public database-based retrospective study
Source: PeerJ. 2021 May 5;9:e11319. doi: 10.7717/peerj.11319 (PMC8106393; doi:10.7717/peerj.11319)
Supplement: Table S1 [file peerj-09-11319-s001.docx]

Table S1. Go analysis of 303 overlapping DEGs.

| Category | Term | Count | FDR |
| --- | --- | --- | --- |
| MF | receptor activity | 19 | <0.001 |
|  | transmembrane signaling receptor activity | 17 | <0.001 |
|  | chemokine activity | 8 | 0.014 |
|  | C-C chemokine receptor activity | 5 | 0.036 |
| CC | integral component of plasma membrane | 71 | <0.001 |
|  | external side of plasma membrane | 29 | <0.001 |
|  | extracellular region | 69 | <0.001 |
|  | extracellular space | 55 | <0.001 |
|  | plasma membrane | 111 | <0.001 |
|  | extracellular exosome | 76 | 0.004 |
| BP | immune response | 49 | <0.001 |
|  | inflammatory response | 40 | <0.001 |
|  | adaptive immune response | 24 | <0.001 |
|  | innate immune response | 35 | <0.001 |
|  | cell surface receptor signaling pathway | 27 | <0.001 |
|  | chemotaxis | 18 | <0.001 |
|  | regulation of immune response | 20 | <0.001 |
|  | positive regulation of T cell proliferation | 13 | <0.001 |
|  | signal transduction | 50 | <0.001 |
|  | chemokine-mediated signaling pathway | 13 | <0.001 |
|  | cellular defense response | 12 | <0.001 |
|  | B cell receptor signaling pathway | 11 | <0.001 |
|  | positive regulation of ERK1 and ERK2 cascade | 16 | <0.001 |
|  | cell-cell signaling | 19 | <0.001 |
|  | dendritic cell chemotaxis | 7 | <0.001 |
|  | positive regulation of cytosolic calcium ion concentration | 14 | <0.001 |
|  | T cell costimulation | 10 | 0.007 |
|  | leukocyte migration | 12 | 0.007 |
|  | positive regulation of interferon-gamma production | 8 | 0.014 |
|  | cellular response to lipopolysaccharide | 11 | 0.025 |
|  | negative regulation of T cell proliferation | 7 | 0.043 |
|  | positive regulation of interleukin-2 biosynthetic process | 5 | 0.049 |
|  | receptor activity | 19 | <0.001 |
|  | transmembrane signaling receptor activity | 17 | <0.001 |
|  | chemokine activity | 8 | 0.014 |
