# Supplementary figures and images for: Identification of P2RY13 as an immune-related prognostic biomarker in lung adenocarcinoma: A public database-based retrospective study
Source: PeerJ. 2021 May 5;9:e11319. doi: 10.7717/peerj.11319 (PMC8106393; doi:10.7717/peerj.11319)

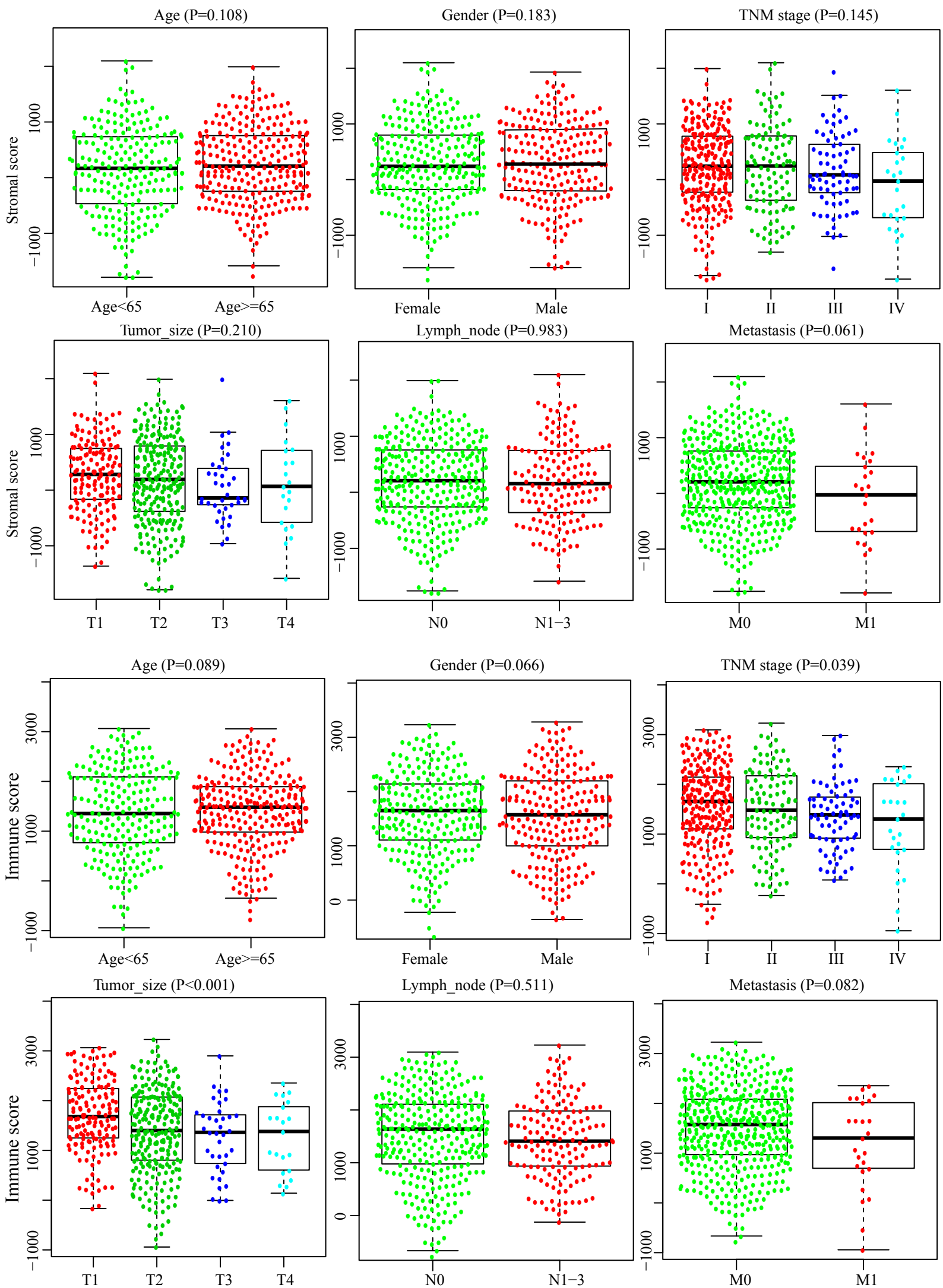

Supplement: Figure S1 — The data were compared with T-test. P < 0.05 was set as the threshold. [file peerj-09-11319-s002.pdf]

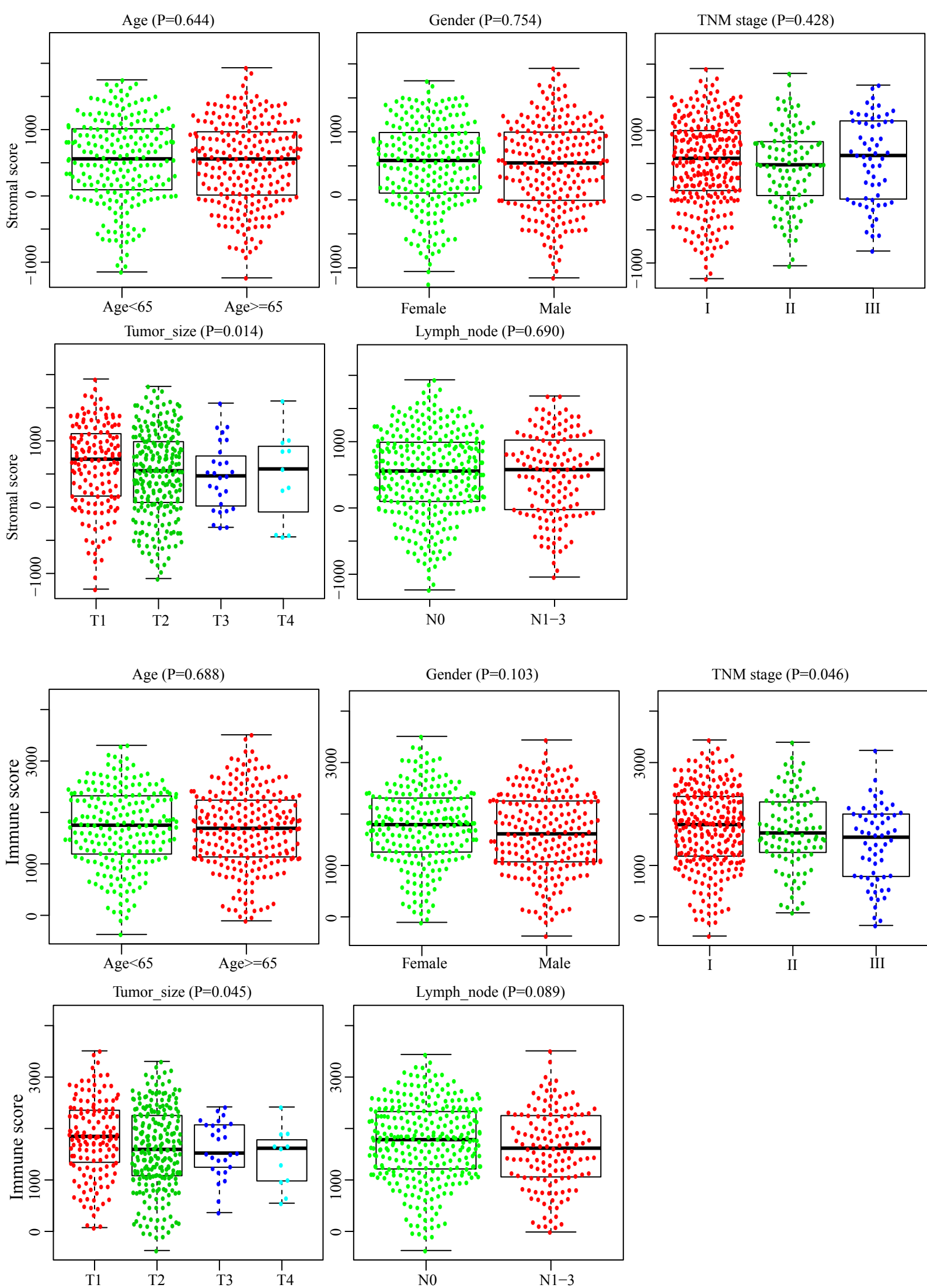

Supplement: Figure S2 — The data were compared with T-test. P < 0.05 was set as the threshold. [file peerj-09-11319-s003.pdf]

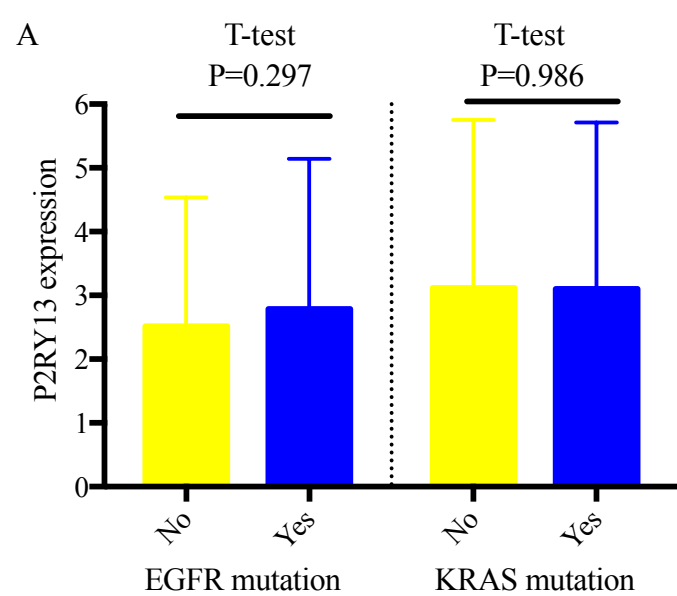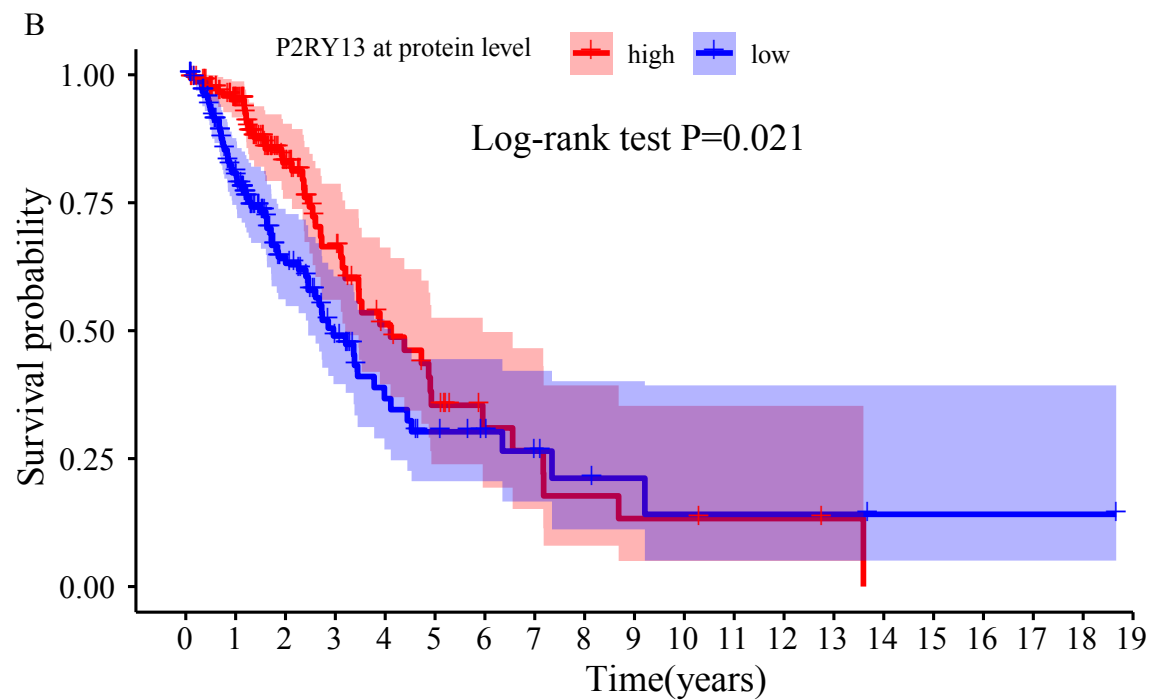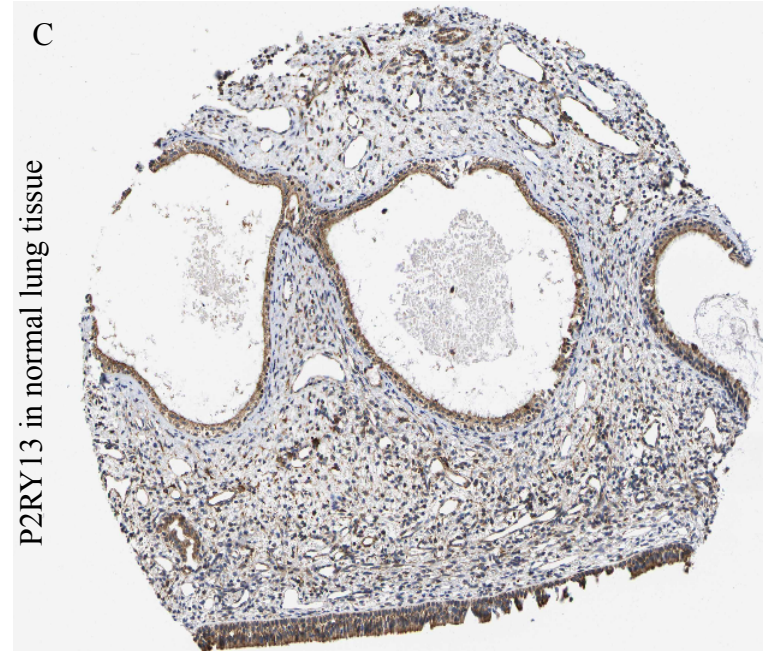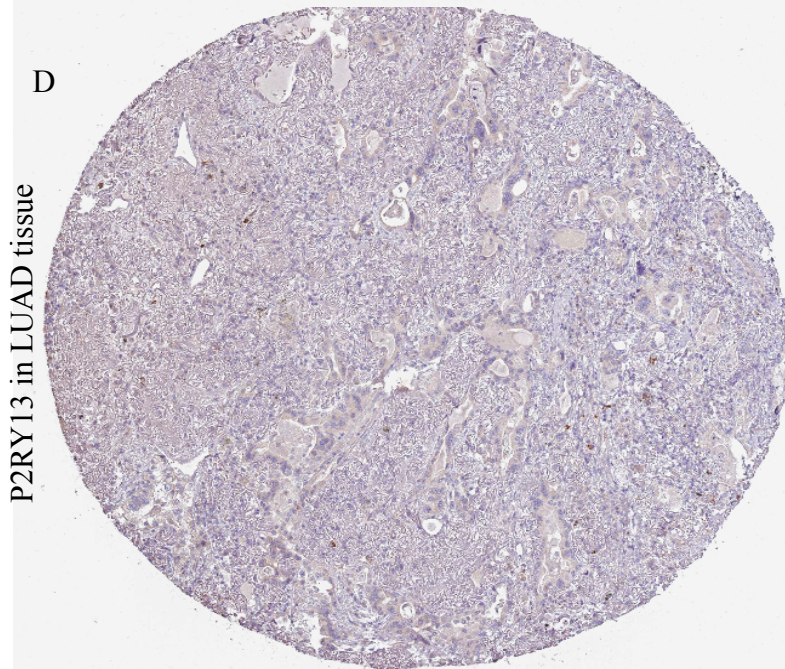

Supplement: Figure S3 — (A) Comparison of P2RY13 expression between patients with and without EGFR/KRAS mutation. The data were presented as mean ± SD (standard deviation), and compared with T-test. (B) The prognostic value of P2RY13 at protein level. The survival difference was compared with log-rank test. P < 0.05 was set as the threshold. (C–D) The results of Immunohistochemistry showed the representative protein expression of P2RY13 in LUAD tissues was down-regulated. [file peerj-09-11319-s004.pdf]

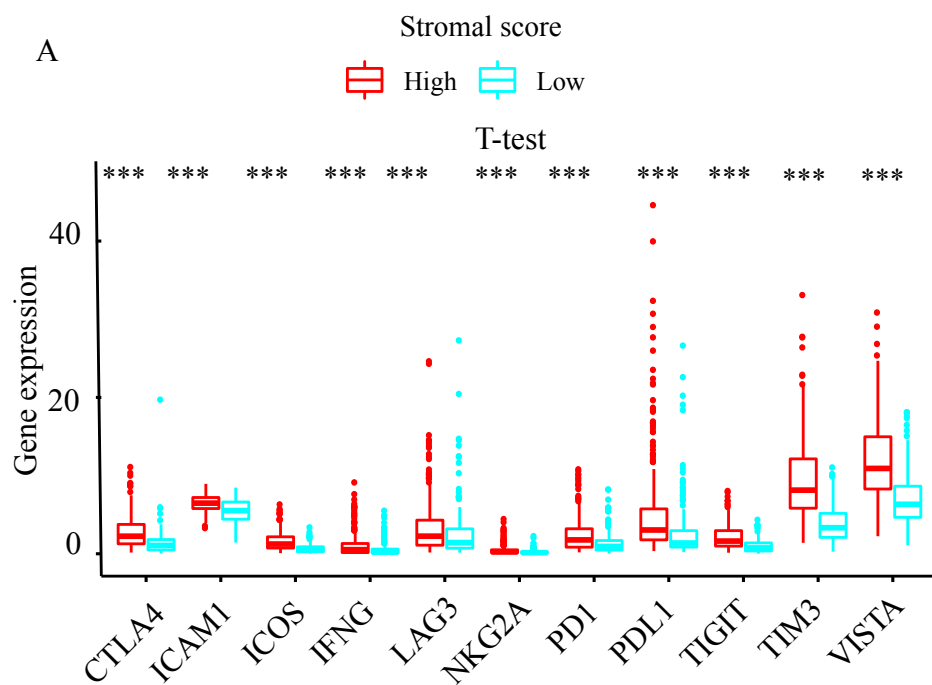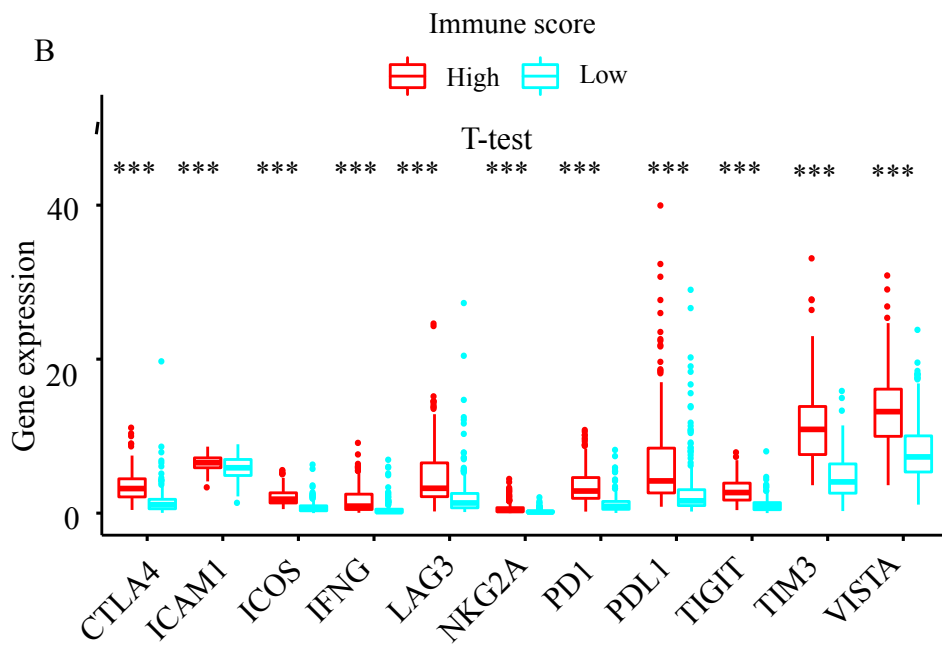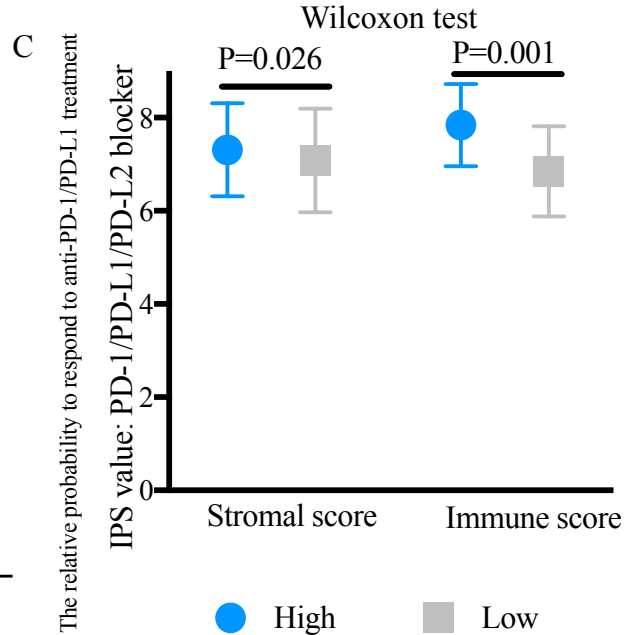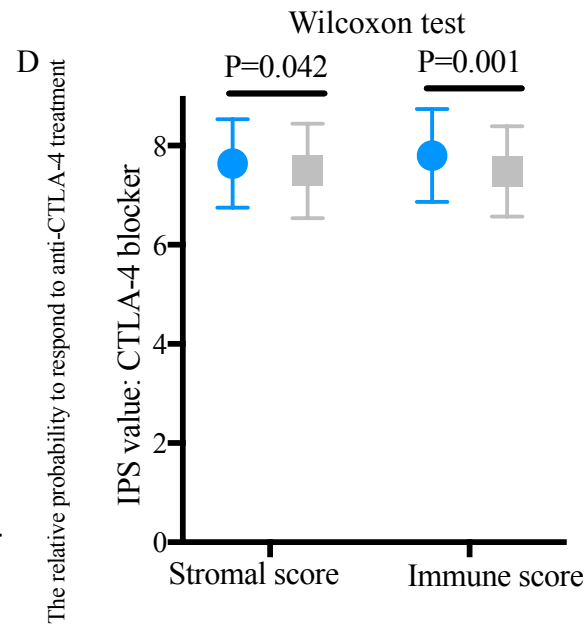

Supplement: Figure S4 — The expression of immunomodulators (A) between high- and low- stromal score group, (B) between high- and low- immune score group. The data were presented as mean± SD (standard deviation), and compared with T-test. (C–D) The relative probabilities to respond to anti-PD-1/PD-L1 and anti-CTLA-4 treatment in LUAD patients with high and low stromal/immune expression. The data were presented as mean ± SD (standard deviation), and compared with Wilcoxon test. P < 0.05 was set as the threshold. [file peerj-09-11319-s005.pdf]
